# Metaphoric Reference: An Eye Movement Analysis of Spanish–English and English–Spanish Bilingual Readers

**DOI:** 10.3389/fpsyg.2016.00439

**Published:** 2016-03-29

**Authors:** Roberto R. Heredia, Anna B. Cieślicka

**Affiliations:** Cognitive Neuroscience Laboratory, Department of Psychology and Communication, Texas A&M International UniversityLaredo, TX, USA

**Keywords:** anaphoric metaphor, bilingual metaphor, language dominance, metaphoric reference, referential metaphor

## Abstract

This study examines the processing of metaphoric reference by bilingual speakers. English dominant, Spanish dominant, and balanced bilinguals read passages in English biasing either a figurative (e.g., describing a *weak and soft fighter that always lost and everyone hated*) or a literal (e.g., describing a *donut and bakery shop that made delicious pastries*) meaning of a critical metaphoric referential description (e.g., “creampuff”). We recorded the eye movements (first fixation, gaze duration, go-past duration, and total reading time) for the critical region, which was a metaphoric referential description in each passage. The results revealed that literal vs. figurative meaning activation was modulated by language dominance, where Spanish dominant bilinguals were more likely to access the literal meaning, and English dominant and balanced bilinguals had access to both the literal and figurative meanings of the metaphoric referential description. Overall, there was a general tendency for the literal interpretation to be more active, as revealed by shorter reading times for the metaphoric reference used literally, in comparison to when it was used figuratively. Results are interpreted in terms of the Graded Salience Hypothesis (Giora, 2002, 2003) and the Literal Salience Model (Cieślicka, 2006, 2015).

## Metaphoric Reference: An Eye Movement Analysis Of Bilingual Readers

This study examines the comprehension of literal and non-literal meanings of a metaphoric description by Spanish–English and English–Spanish bilinguals at the discourse level. As a way to introduce the topic of this paper, consider the interpretation of a somewhat novel non-literal expression by a bilingual speaker as a professor remarked, “Back then in the late 1980s, I used to write poetry. Now I only write sleeping pills." To which a bilingual student responded, “You mean prescription drugs?" The professor’s intended meaning (i.e., non-literal interpretation) was that he used to write fun and interesting stuff, and now he was writing books that were making people fall asleep. However, the student interpreted the expression literally as “sleeping pills," which are typically related to drugs used to induce sleeping. “Sleeping pills," in this case, is an example of a metaphoric referential description or an anaphoric metaphor (e.g., Gibbs, 1990; Onishi and Murphy, 1993; Budiu and Anderson, 2002; Stewart and Heredia, 2002; Almor et al., 2007; Heredia and Muñoz, 2015).

Anaphoric metaphors, or metaphoric referential descriptions, contrast with the conventionalized nominal metaphor of the form *A is B* (e.g., “books are sleeping pills”), where “books” is the *tenor* or *subject* (A) of the metaphor; “sleeping pills” is the *vehicle* that provides attributes characterizing the topic, and the *ground* is the basis on which it is possible to infer a relationship between the subject and the vehicle. Unlike in the nominal metaphor, the subject and vehicle in metaphoric referential descriptions occur apart from each other. The vehicle (i.e., “sleeping pills”) is used as the reference for the previously mentioned or implied subject (“books”). Thus, understanding referential descriptions requires that readers or listeners establish a connection between the anaphoric metaphor (“sleeping pills”) and its antecedent (“books”) found elsewhere in the sentence. This reactivation of the antecedent from the metaphoric reference, as argued by Gibbs (1990), Onishi and Murphy (1993), and Almor et al. (2007) requires additional inferential processes to understand the intended meaning (but see Heredia and Muñoz, 2015; see also Stewart and Heredia, 2002).

Although our knowledge about how bilinguals comprehend non-literal language in such domains as idiomatic expressions is on the rise (see for example, Heredia and Cieślicka, 2015), very few studies have looked at how bilingual speakers comprehend metaphoric referential descriptions in their second language (L2). The purpose of the present study is to look at early- vs. late-lexical processing of literal vs. non-literal language interpretation using eye movement recordings (Rayner, 2009), and to assess the effects of language dominance in Spanish–English and English–Spanish bilinguals (cf. Johnson and Rosano, 1993; Heredia, 1997; Cieślicka et al., 2014; cf. Cieślicka, 2015).

### Models of Metaphoric Processing

Two general theoretical models have been traditionally proposed in the monolingual literature to account for how metaphoric expressions are comprehended. These models have been extended to explain bilingual figurative language processing (e.g., Matlock and Heredia, 2002; Heredia and Muñoz, 2015; Vaid et al., 2015). The *Direct Access Model* assumes that during the course of comprehending a metaphoric expression, its intended or non-literal interpretation may be accessed directly “without first requiring an initial literal interpretation computed and rejected” (Blasko and Connine, 1993, p. 295; Glucksberg, 2001; see also Vaid et al., 2015). Although a literal interpretation may be temporarily available to construct the non-literal meaning of a metaphor, it is neither obligatory nor required before the metaphoric comprehension begins (Blasko and Connine, 1993, p. 295). For the *Indirect Processing Model* (Searle, 1979; Swinney and Osterhout, 1990), the metaphor’s literal interpretation is obligatory, and only if the literal interpretation is defective or does not fit the context, a search for a non-literal interpretation is initiated (Heredia and Muñoz, 2015). Although the empirical evidence supports the Direct Access Model, studies utilizing more sensitive online methods measuring language processing in real time have unequivocally supported the Indirect Processing Model (see for example, Janus and Bever, 1985; Swinney and Osterhout, 1990). However, other studies (e.g., Blasko and Connine, 1993; Heredia and Muñoz, 2015) utilizing similar methodologies, have reported findings difficult to reconcile by both theoretical frameworks.

A third model of metaphoric processing is the *Graded Salience Hypothesis* [GSH; Giora (2003; see also, Cieślicka, 2006, 2015)]. Briefly, the GSH assumes that metaphoric expressions are best understood depending on which meaning (i.e., literal vs. figurative) is more salient. *Salient meanings* are readily accessible, excitable, and influenced by such factors as word frequency, familiarity, conventionality, and prototypicality/stereotypicality. *Non-salient meanings*, on the other hand, are less frequently used, less familiar, and would take longer to be triggered, requiring extra-inferential processes (Giora, 2003, p. 491). Thus, for an English native speaker, the figurative meaning of a highly familiar metaphor such as *loneliness is a desert* would be highly salient and readily interpretable as relating to a feeling of isolation. The literal meaning, related to “sand” or “dryness,” would be less salient, resulting in longer response times. The opposite would be true for low familiarity metaphoric expressions. In this case, the literal meaning of the metaphoric expression would be more salient (see for example, Blasko and Connine, 1993), thus allowing explaining the literal interpretation presented in the introductory example, where the student in question understood “sleeping pills” as a narcotic prescription medication.

Although the GSH has not been applied to bilingualism or L2 metaphoric processing, it could be argued that *meaning saliency* (i.e., a metaphor’s literal vs. non-literal interpretation) could also be influenced by L2/bilingual factors, such as language dominance (Heredia, 1997; Dunn and Fox Tree, 2009; Heredia and Cieślicka, 2014), language proficiency, linguistic environment (i.e., bilingual vs. monolingual community), first language (L1), and L2 age of acquisition, and acquisition context (i.e., whether L2 was acquired at home vs. school). Thus, for a bilingual speaker who is highly proficient and dominant in the L2, figurative interpretations of highly familiar metaphoric expressions, like for monolinguals, would be highly salient and readily accessible (e.g., Heredia and Muñoz, 2015). However, for less proficient learners of an L2, literal reading of a metaphoric expression would take precedence and become more salient (see for example, *Literal Salient Model*; Cieślicka, 2006, 2015).

### Bilingual Metaphoric Language Processing

How do bilinguals comprehend metaphoric expressions? Although limited, experimental evidence seems to suggest that, like monolinguals, bilingual speakers might be able to directly access the intended (figurative) meaning of a metaphoric expression, as predicted by the Direct Access Model. In one of the first studies, Nelson (1992) investigated memory for metaphor by non-fluent bilinguals. Spanish–English and French–English bilinguals translated metaphorical and literal expressions from Spanish (e.g., *Un árbol es un paraguas or un árbol es fuerte*) or French (*Un arbre est un parapluie* or *Un arbre est fort*) into English (*A tree is an umbrella* or *A tree is strong*). For this condition, participants were explicitly instructed to translate the figurative or literal meaning of a metaphoric expression into English, the L2. In another experimental condition, bilinguals received a list of metaphors and literal expressions and were asked to simply translate them into L2. This condition did not require participants to consciously trigger the figurative interpretation of the metaphoric expression. A cued-recall task (e.g., *A tree is__*) in Spanish or French was used to measure retention. Results revealed that translating the figurative meaning of a metaphor into L2 significantly improved retrieval, relative to translating the literal meaning of a metaphoric expression. More significant, however, was the finding that translating the figurative meaning of the metaphor did not produce better recall than the condition in which participants were simply asked to translate a metaphoric expression into L2. These results were interpreted as suggesting that the processing of the figurative meaning of a metaphor is *automatic* and that the processing of the literal interpretation of a metaphor is not obligatory (see Glucksberg and Keysar, 1990; see also Vaid et al., 2015). In fact, asking participants to interpret the literal meaning of a metaphor actually *interfered* with normal processing, thus resulting in poor recall performance (Nelson, 1992). By and large, studies interpreted as supporting the Direct Access Model involve nominal metaphors of the type *A is B*.

More recently, Heredia and Muñoz (2015) utilized the cross-modal naming task (Swinney, 1979; Blasko and Connine, 1993; Tabossi, 1996; Heredia and Blumentritt, 2002; Cieślicka, 2006; Cieślicka and Heredia, 2016) to explore the temporal course of meaning activation (i.e., literal vs. figurative) of metaphoric referential descriptions. Highly fluent bilinguals in English, the L2, from a predominantly English-Speaking area (Experiment 1) and Spanish–English/English–Spanish bilinguals from a bilingual community (Experiment 2) listened to story passages as the one described in (1) below.

(1) Stu went to see the Saturday night fights. There was one fighter that Stu hated. This guy always lost. Just as the match was about to start, Stu went to get some snacks. He stood in line for 10 min. When he returned, the bout had been canceled. “What happened?” Stu asked a friend. His friend replied, “Aw, the creampuff_[__∗1]_ didn’t even show_[∗2]_ up, I can’t believe it!”

As participants listened to the passage, a visual target appeared either at metaphor offset for Experiments 1 and 2 (position *1 depicted by subscripts), 1000 ms (Experiment 1), or 300 ms (Experiment 2) after metaphor offset (position *2). The visually presented target words were either related literally (e.g., “pastry”) or non-literally (e.g., “boxer”) to the metaphoric referential description (“creampuff”), or they were unrelated controls (“pirate” and “camel”).

At issue was whether “creampuff,” the vehicle of the metaphor (i.e., the anaphor), would (re)activate its antecedent (“fighter,” the non-literal meaning) and its literal interpretation (“pastry”). The priming effect was taken as a measurement of meaning (literal vs. non-literal) activation, or the extent to which a particular meaning is activated relative to its control. Briefly, the priming effect refers to the robust finding whereby response to a target (e.g., “bread”) is faster when preceded by a related (e.g., “butter”) than an unrelated word (e.g., “mirror”).

Results from Experiment 1 showed that bilinguals living in a predominantly English environment were able to (re)activate the antecedent (“boxer”) immediately after the metaphoric referential description (Position 1). In contrast, there was no evidence of literal meaning activation. However, at 1000 ms, only literal activation was evident. Although bilinguals had direct access to the non-literal interpretation, as in Stewart and Heredia’s (2002) English monolingual speakers, activation of the literal interpretation suggested the literal interpretation remained as a possibility for bilinguals, even 1000 ms after they had accurately resolved the linguistic ambiguity (cf. Blasko and Connine, 1993). Experiment 2 indicated that, regardless of the target position, both the literal and non-literal meanings of the metaphoric referential description were equally accessible, particularly 300 ms after metaphor offset. However, it appeared that the literal interpretation was relatively more active. Heredia and Muñoz’s (2015) results were more consistent with the GSH suggesting that bilinguals, like native speakers of English, might have direct access to the metaphoric figurative interpretation. However, the overall evidence from these two experiments points to the possibility that literal meanings are more strongly coded or more salient in the bilingual’s lexicon (e.g., Cieślicka, 2006, 2015; cf. Vaid et al., 2015).

### The Present Study

How do bilinguals store and process metaphoric expressions? The purpose of the present study is to further investigate the on-line comprehension of referential metaphoric expressions using eye movement recordings, which provide temporally precise measures, to capture fine-grain differences, if any, between the literal and figurative interpretations of a metaphoric expression. If temporal differences (i.e., early vs. late processing stages) exist between literal and figurative meaning interpretations, as predicted by Direct and Indirect Processing Models, eye movement recordings reflecting early (e.g., first fixation duration or duration of the very first fixation on a word), and late stages (e.g., total reading time or the sum of all fixation durations) of lexical processing (e.g., Rayner, 1998, 2009; Raney and Bovee, 2015; Whitford et al., 2015) will reveal these processing differences. It may very well be the case that findings in which figurative meanings take precedence over literal interpretations (e.g., Stewart and Heredia, 2002; Heredia and Muñoz, 2015, Experiment 1) reflect late stages of lexical processing or semantic integration. Another aim of the present study is to investigate the effects of language dominance (i.e., fluency, lexical and syntactic knowledge, ease of accessibility; Dunn and Fox Tree, 2009) on literal and figurative meaning activation in metaphoric processing. Previous studies have only looked at bilingual directionality in relation to the bilingual’s L1 (Spanish or English) and L2 (English or Spanish). In fact, bilingual models of word recognition (e.g., Kroll and Stewart, 1994) hypothesize retrieval differences between L1–L2 and L2–L1. However, language dominance may be a better predictor of lexical access, regardless of bilingual directionality (e.g., Heredia and Muñoz, 2015).

Participants in the present study read short passages such as (1) above, describing a weak boxer being referred to as a “creampuff” (the metaphoric condition). For the literal condition, unlike the original studies (e.g., Gibbs, 1990; Onishi and Murphy, 1993), the same target was utilized. However, the preceding context was biased toward the literal interpretation of the critical target (e.g., “creampuff”), as in (2) below:

(2) Stu and his buddy went to the donut shop. There was a baker who made delicious pastries. Just before they ordered, Stu went to the bathroom. When he came back his buddy was outside the bakery shop. “What happened?” Stu asked, and his friend replied, “Aw, the creampuff wasn’t even that good! I can’t believe it.”

The present study differs from Gibbs (1990) and Onishi and Murphy (1993), in that participants are presented with full passages (as in 1 and 2 above), instead of seeing each passage line by line. Another notable difference is that, for both literal and metaphoric conditions, the same critical target (“creampuff”) is used, thus controlling for word level effects. At issue is whether the figurative interpretation of the metaphoric referential description (“creampuff” as a weak boxer in passage 1) is faster and more readily accessible during early stages of reading comprehension, as predicted by the Direct Access Model, or whether the literal sense (“creampuff” as a pastry in passage 2) has precedence over the figurative interpretation. In this case, eye movement recordings for early stages of reading comprehension (e.g., first fixation duration) will reveal faster reading times for the literal interpretation. However, the figurative meaning interpretation of the metaphoric reference should be faster during the late stages, as reflected by such eye movement measures as, for example, total reading time of lexical processing. In relation to language dominance, English dominant bilinguals are expected to have direct access to the figurative interpretation of the metaphoric referential description, as in Stewart and Heredia (2002) and Heredia and Muñoz (2015). However, Spanish dominant bilinguals are likely to conform to a language processing configuration in which the literal meaning is more salient during both early and late stages of reading comprehension, as predicted by the GSH (Giora, 2003), and Cieślicka’s (2006, 2015) Literal Salience Model.

To our knowledge, no eye-tracking studies have explored the effects of language dominance in the comprehension of metaphoric referential expressions or nominal metaphor by bilinguals. At the monolingual level, there is only one study using eye movements to explore processing differences between literal and non-literal interpretation in metaphoric processing. Inhoff et al. (1984) explored the effects of prior context (biased vs. non-biased) in the comprehension of nominal metaphors. Participants read passages that were either literally biased, (3) *in the back of the barn, the farmer’s youngest child gathered pebbles and skipped them deftly across a puddle by the chicken coop. He knew that he was supposed to be feeding the animals but he kept on flicking at the birds. The hens clucked noisily;* literally non-biased (4) *In the back of the barn, the hens clucked noisily;* (5) non-literally biased, *at a meeting of the women’s club the youngest member requested the floor and brought up the issue of supporting the equal rights amendment. The importance of the issue outweighed her discomfort in speaking before the group. They reacted as she expected. The hens clucked noisily*; and non-literally non-biased, (6) *At a meeting of the women’s club, the hens clucked noisily*. Results from the Inhoff et al. (1984) study revealed no differences between literal and non-literal targets under biased contextual conditions. Under non-biased contextual conditions, literal targets were recognized more quickly than non-literal targets. It should be noted, however, that Inhoff et al.’s (1984) eye movement measures (e.g., total reading time and sentence reading time) reflected late stages of reading comprehension, and it is unclear if their results can be generalized to early processing stages of reading comprehension. To sum up, we ask the following questions: (1) what are the effects of language dominance in metaphoric processing? (2) Will there be differences between early and late stages of reading comprehension between literal and figurative interpretations of metaphoric referential expressions among bilinguals?

## Materials and Methods

### Participants

Forty Spanish-English and 32 English–Spanish bilinguals (female = 55, male = 17) from the psychology subject pool at Texas A&M International University participated in the experiment. Participants volunteered or received class credit as a partial class requirement. Four participants were excluded from further analysis due to computer errors; one other participant was excluded because the language questionnaire was unavailable. Language dominance was assessed by Dunn and Fox Tree’s (2009) Bilingual Dominance Scale. Based on their aggregated scores, 20 participants were classified as “balanced” (*M* = -1.05, *SD* = 2.87), 36 as English dominant (*M* = 14.9, *SD* = 4.80), and 16 as Spanish dominant (*M* = -14.4, *SD* = 6.32). The participant’s mean age was 23.3 years (*SD* = 4.02, *range* = 19–38). Following Vaid et al. (2015), a composite language-proficiency score was created that included speaking, reading, understanding, and writing Spanish (*M* = 5.56, *SD* = 1.25) and English (*M* = 6.42, *SD* = 0.9). A dependent *t-test* revealed that participant’s proficiency scores were higher for English, [*t*(71) = 4.77, *p <* 0.01].

**Table 1** summarizes participants’ responses to language performance measures broken down into bilingual directionality (English–Spanish vs. Spanish–English) and English and Spanish. Both bilingual groups (English–Spanish vs. Spanish–English) reported similar formal education in Spanish and English. Likewise, both groups reported similar incidences of language mixing or code-switching (see for example, Heredia and Altarriba, 2001), where both languages are used simultaneously. Mean self-ratings for English usage on a typical day and language fluency in English and Spanish for speaking, reading, understanding, and writing show that bilinguals rated English as the most frequently used and more proficient language.

**Table 1:** Language background information for the bilingual sample.

	English–Spanish	Spanish–English
Age	24.1 (5.29)	22.5 (2.50)
Mean age L2 learned	5.81 (2.30)	6.68 (3.92)
Mean years of schooling in Spanish	4.03 (6.07)	6.48 (6.00)
Mean years of schooling in English	13.6 (5.54)	11.2 (5.68)
Mean language mixing Ratings	4.75 (2.03)	5.18 (1.87)
**Mean Self-Ratings**	**English**	**Spanish**
Language usage	6.11 (1.32)	5.25 (1.68)**
Speaking	6.23 (1.07)	5.68 (1.1)**
Reading	6.46 (0.948)	5.54 (1.41)**
Understanding	6.61 (0.707)	6.11 (1.33) **
Writing	6.35 (1.14)	4.86 (1.71)**

Values in parentheses represent SDs; **p < 0.01; Self-ratings (1 = Not fluent, 7 = Very fluent); Language mixing (1 = I Never mix languages, 7 = I Mix languages all the time).

**Table 2** summarizes correlations (*r*) between variables typically used to measure language proficiency and language dominance. It is noteworthy that language usage and language proficiency are significantly correlated with language dominance. The overall pattern, depicted in **Table 2**, suggests that, as language use, language proficiency, and language dominance increase for one language, the other language decreases in the same indicators. Another important finding is that language mixing is positively correlated with Spanish and English proficiency. More notable, however, is the moderate correlation between proficiency and dominance for both languages and the strong negative relationship between language dominance in Spanish and English.

**Table 2:** Summary of intercorrelations for scores on language variables.

Variables	AgeE	AgeS	S_sch	E_sch	Mix	S_use	E_Use	S_prof	E_prof	E_dom
AgeS	-0.09									
S_sch	0.30*	-0.15								
E_sch	-0.22	0.15	0.09							
Mix	0.01	-0.24*	-0.06	0.05						
S_use	0.35**	-0.38**	0.24*	-0.06	0.57**					
E_use	-0.36**	0.148	-0.42**	0.20	0.23	-0.12				
S_prof	0.38**	-0.35**	0.19	-0.18	0.27*	0.65**	-0.21			
E_prof	-0.33**	0.03	-0.34**	0.22	0.24*	-0.07	0.68**	-0.01		
E_dom	-0.70**	0.30*	-0.24*	0.32**	-0.03	-0.41**	0.57**	-0.42**	0.50**	
S_dom	0.60**	-0.47**	0.33**	-0.28*	0.04	0.41**	-0.54**	0.480**	-0.42**	-0.83**

AgeE/S, age learned English and Spanish; S_sch/E_sch, years of schooling in Spanish/English; Mix, code-switching; S_use/E_use, Spanish/English usage in a typical day; S_prof/E_prof, Spanish/English proficiency; E_dom/S_dom, English/Spanish dominance; *p < 0.05. **p < 0.01.

### Materials and Design

Stimuli consisted of 40 short passages, as described in 1–2 in the introduction, each a brief exchange between two persons describing a mutually known person or thing. Figurative-biased passages (see passage 1 above) were taken directly from Stewart and Heredia (2002) and Heredia and Muñoz (2015). Literal-biased passages were created following the same format as the figurative-biased ones. For both passages, the target item (e.g., “creampuff”) appeared in the penultimate or last sentence, and it was preceded by a description of a very weak boxer (figurative condition) or a pastry or donut that was not that good after all (literal condition). For both conditions, sentences containing the critical target were constructed as similarly as possible (e.g., figurative: “Aw, the creampuff didn’t even show up, I can’t believe it” vs. literal: “Aw, the creampuff wasn’t even that good! I can’t believe it”). Average number of words for the metaphorical (*M* = 64.1, *SD* = 10.4) and literal passages (*M* = 61.6, *SD* = 11.5) was the same, *t*(78) = 1.03, *p* = 0.31; likewise, the average number of words before the target location between literal (*M* = 50.2, *SD* = 10.7) and figurative (*M* = 53.3, *SD* = 10.0) passages was comparable, *t*(78) = 1.33, *p* = 0.19. Average number of words for the target location within each passage was 54.3 (*SD* = 9.95), for the figurative and 51.2 (*SD* = 10.7) for the literal conditions. The 3.1 word difference between the two passages was not statistically significant, *t*(78) = 1.33, *p* = 0.19.

Two lists were required to counterbalance each critical target within a passage. Each list contained 20 figurative- and 20 literal-biased passages. Lists were constructed in such a way that no passage with the same target (literal or non-literal) was repeated within a list. Stimuli assignment was between lists using an ABBA BAAB counterbalancing procedure. Forty passages were used as fillers. These passages were taken from Stewart and Heredia (2002) and Heredia and Muñoz (2015). Fillers were matched to the experimental stimuli on format and number of sentences and contained no metaphorical reference or any hint of figurative language (e.g., *Holy wanted to be the first female admitted to the male-only military academy. On her way to the academy, she noticed she didn’t have her teddy bear. “Stop!” she yelled to the driver. “You have to go back, I forgot my teddy bear and I need it to keep me company and to support me.” The driver responded very angrily, “Forget it, I am not going back!”*) The 80 passages were combined in a pseudo-random order, which imposed the constraint that no more than three experimental conditions occurred consecutively. Five additional filler passages served as practice trials. After each passage, participants responded to a true/false comprehension question. There were a total of 80 questions.

### Design

The critical values measured were first fixation duration (the length of time the eyes spend on the target word the very first time they land on it), gaze duration (the sum of the duration of all fixations made on the word prior to exiting the word), total reading time (the sum of all fixation durations made on the target word, including re-reading), and go-past time (the sum of all fixation durations, which starts with the first fixation on the word up to the time the eyes fixate to the right of the word), both for the figurative (passage 1) and literal (passage 2) targets. The design conformed to a 2 (target type: figurative vs. literal) × 3 (language dominance: Spanish vs. English vs. balanced) mixed factorial design, with target type as a within-subjects factor, and language dominance as a between-subjects variable.

### Procedure

Upon arrival to the laboratory, participants completed a pencil and paper consent form. They were then instructed to sit comfortably, so that they were able to position their chin on a chinrest and maintain stability. Visual calibrations were conducted to ensure that the eye tracker was accurately recording the participants’ eye movements. The visual recording was monocular, where the eye tracker recorded the right eye.

Experimental stimuli were presented using SR-Experiment Builder running on Windows OS 7, and the EyeLink 1000 eye tracker was connected to a dedicated host computer running on DOS. Participants were randomly assigned to one of the two experimental lists. They were seated approximately 55 cm from the monitor, with their head supported by a chinrest. Following eye-tracking calibration, the instructions were displayed on the computer screen. Participants were asked to read each passage shown on the screen and to answer a comprehension question that followed. Comprehension questions were of the yes/no type and were randomly displayed after every few sentences to ensure participants were attending to the passages. Participants were provided with 10 practice trials to familiarize them with the experimental procedure. Each trial started with a black fixation point appearing on the left of the screen, where the first word of the passage would appear. Participants were instructed to focus their eyes on the fixation point and to press the designated button on a Microsoft SideWinder Plug and Play Game Pad (Model GP5) game controller device in order to trigger the sentence display. After reading each passage, participants pressed the game controller button to advance to the next trial. Passages were displayed in black Times New Roman 20 font against a white background. The eye monitoring session lasted approximately 30 min. Following the experiment, participants completed the Bilingual Dominance Scale language background questionnaire and were debriefed as to the purpose of the experiment. The experimental protocol was approved by the Texas A&M International University Institutional Review Board (IRB).

## Results

Participants’ responses to comprehension questions were analyzed for accuracy. All participants answered the comprehension questions with an accuracy above 90%, with the exception of one participant whose accuracy was 85%. Responses were normally distributed across the two experimental conditions. Data from four participants were excluded due to computer errors. Data from one additional participant were excluded due to an incomplete language questionnaire. The data were analyzed using linear mixed effects models (LME) using IBM SPSS V.20, mixed linear models procedure, with fixed (i.e., independent variables; target type and language dominance) and random effects (i.e., items and subjects). Analyses were conducted on both early (first fixation and gaze duration) and late (total reading time and go past duration) stage reading measures (Rayner, 1998). For all the measures, percentage of data removed and percentage of targets skipped as a function of the experimental conditions are provided.

### First Fixation Duration

**FIGURE 1:**
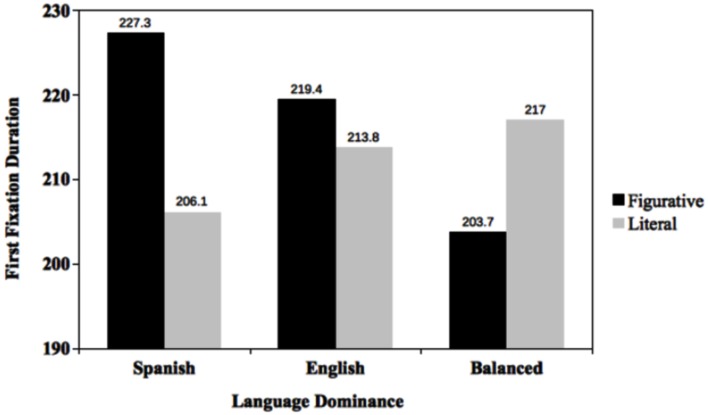
**First fixation duration for figurative- and literal-biased targets as a function of language dominance**.

A total of 3.4% of the data were removed because fixation durations were less than 100 ms (Libben and Titone, 2009). The LME 2 × 3 analysis yielded a statistically reliable interaction between language dominance as a function of target type, *F*(2,1482.2) = 5.01, *p* < 0.01. **Figure 1** summarizes the interaction. Follow up *F-tests* show that Spanish dominant bilinguals read literal targets faster than figurative ones, *F*(2,595,0) = 6.0, *p* < 0.05. However, English dominant bilinguals were equally fast in reading both literal and figurative targets. Balanced bilinguals, on the other hand, were faster in reading figurative than literal targets; however, the reading differences were not significant, *F*(2,437.6) = 2.93, *p* = 0.09. No other effects reached significance.

The eye movement recordings showed a high proportion of participants skipping the target region. A higher rate of literal targets (*M* = 0.474, *SE* = 0.029) was skipped in comparison to figurative targets (*M* = 0.380, *SE* = 0.029), *F*(1,83.1) = 7.74, *p* < 0.01. Skipping a word during reading is reflective of the word’s predictability, or the participant’s ability to anticipate the target word based on the preceding contextual constraints (e.g., Ehrlich and Rayner, 1981; Balota et al., 1985; Rayner and Well, 1996; Rayner et al., 2011). It is likely that literal targets, in this case, were easy to predict (and hence skipped) based on the preceding contextual information that was readily integrated, as opposed to the figurative target that required additional inferential processes (Giora, 2002; see also Onishi and Murphy, 1993; Almor et al., 2007).

### Gaze Duration

A total of 2.5% of the data were removed because gaze durations were less than 100 ms. The two-way analysis revealed a significant main effect of target type, *F*(1,82.1) = 3.84, *p* = 0.05. No other effects were reliable. However, it should be noted that, although the interaction was not statistically reliable, gaze duration patterns are similar to the first fixation data, showing the larger differences between literal and figurative targets for Spanish dominant bilinguals. In relation to target type, literal targets (*M* = 261.9, *SE* = 12.03) exhibited shorter reading times than figurative targets (*M* = 289.3, *SE* = 11.8).

In relation to skipping the target region as a function of language dominance and target type, the only reliable main effect was target type, *F*(1,82.9) = 7.56, *p* < 0.01, where the proportion of skipped literal targets (*M* = 0.470, *SE* = 0.029) was higher than figurative targets (*M* = 0.377, *SE* = 0.029).

### Go-Past Duration

A total of 1.7% of the data were removed because fixation durations were less than 100 ms. The two-way analysis revealed a main effect of target type, *F*(1,82.01) = 4.54, *p* < 0.05. Literal targets (*M* = 299.1, *SE* = 15.3) were read faster than figurative targets, (*M* = 335.7, *SE* = 15.0). In regards to skipping the critical target, the two-way interaction revealed a main effect on target type, *F*(1,82.8) = 8.04, *p* < 0.01. No other effects reached significance. The proportion of skipping a literal target (*M* = 0.469, *SE* = 0.029) was larger than for figurative targets (*M* = 0.373, *SE* = 0.029).

### Total Reading Time

A total of 1.8% of the data were removed because fixation durations were less than 100 ms. The two-way analysis revealed a main effect of target type, *F*(1,80.257) = 7.96, *p* < 0.01. No other effects reached statistical significance. Literal targets (*M* = 395.8, *SE* = 25.1) were read faster than figurative targets (*M* = 477.0, *SE* = 25.0). Likewise, the two-way analysis on skipping the target during reading showed a main effect of target type, *F*(1,81.5) = 4.71, *p* < 0.05 No other effects were significant. Literal targets (*M* = 0.190, *SE* = 0.023) exhibited larger proportions of skipping than figurative targets (*M* = 0.133, *SE* = 0.23).

## Discussion

English dominant, Spanish dominant, and balanced bilinguals read passages biasing either a figurative (e.g., describing a *weak and soft fighter that always lost and everyone hated*) or a literal meaning (e.g., describing a *donut and bakery shop that made delicious pastries*) of a metaphoric referential description. For both conditions, we recorded the eye movements for the critical anaphoric reference (e.g., “creampuff” in the boxing/pastry scenarios). We utilized first fixation and gaze durations known for their sensitivity to tap into early stages of lexical processing, and go-past duration and total reading time measurements which reflect late-stage, post-lexical processing. At issue was whether bilinguals would access the figurative interpretation of the metaphoric referential description during the “automatic” or early stages (i.e., first fixation, gaze duration) of reading comprehension, as hypothesized by the Direct Access Model. Alternatively, the literal interpretation should have precedence in the early stages, whereas figurative meaning should emerge in late stages, as posed by the Indirect Processing Model.

The results revealed that, at least for first fixation durations, meaning activation (figurative vs. literal) was moderated by language dominance (see **Figure 1**). Spanish dominant bilinguals yielded shorter reading times for literal than figurative interpretations, suggesting that, based on the overwhelming use of Spanish, the literal representation of the metaphoric expression was more readily accessible. English dominant and balanced bilinguals, on the other hand, have access to both interpretations. This suggests that at early stages of metaphoric processing, English dominant and balanced bilinguals are considering both the literal and figurative interpretations. In relation to existing models of figurative language processing, at least during early stages of bilingual figurative language comprehension, the results are inconsistent with models that predict direct access to the intended (i.e., figurative) meaning only. Neither are the current data consistent with the indirect models predicting literal meaning activation first. Our results indicate that meaning activation is moderated by language dominance and meaning salience (Giora, 2002), in which the literal meaning seems to be more salient than figurative meaning (e.g., Cieślicka, 2006, 2015), especially for Spanish dominant bilinguals.

Gaze duration measurements failed to replicate the findings from first fixation duration. There was a tendency for the literal interpretation to be more active, as revealed by shorter reading times as compared to the figurative interpretation. This finding was generalized throughout both the go-past duration and total reading time measures, as well as skipping rates, in which literal targets were more likely to be skipped than figurative ones. To summarize, gaze duration, go-past duration and total reading time (i.e., measures hypothesized to reflect late stage, post-lexical processing such as semantic integration, revision, problem solving) replicate the original findings reported by Gibbs (1990) and others (Onishi and Murphy, 1993; Budiu and Anderson, 2002; Almor et al., 2007; but see Stewart and Heredia, 2002; Heredia and Muñoz, 2015), showing that the literal interpretations of metaphoric referential descriptions are read faster, as they are easier to process than figurative interpretations. These finding generalize to both monolingual and bilingual experiments investigating reading processes (cf. Inhoff et al., 1984, Experiment 1). These results point to a possible bilingual model of metaphoric processing that is moderated by meaning salience, in which *literal salience* takes precedence over figurative salience (Cieślicka, 2006, 2015).

Taken together, the evidence from the present study qualifies Heredia and Muñoz (2015) results showing that multiple meaning activation occurs, but only for English dominant and balanced bilinguals who are active in English, and only during the early stages of lexical processing. As originally reported by Gibbs (1990), Onishi and Murphy (1993), Budiu and Anderson (2002), and Almor et al. (2007), the present results also show that the literal anaphoric reference to the antecedent (i.e., “creampuff” referring to a pastry) is read faster than metaphoric anaphoric reference (i.e., “creampuff” referring to a coward boxer), but only at late processing stages, as measured by go-past duration and total reading time (cf. Inhoff et al., 1984).

The present results, showing retention of literal meaning at late processing stages, can be addressed within the framework of the *retention hypothesis*, which supplements the GSH (Giora, 2002) and which explains the activation of contextually incompatible meanings in the course of metaphorical comprehension. According to the retention hypothesis, contextually incompatible meanings accessed initially on account of their salience may, subsequently, be either maintained or suppressed, depending on their contribution to the utterance interpretation. Accordingly, inappropriate meanings which are conducive to the compatible interpretation are retained, whereas meanings conflicting with the compatible meaning are discarded. Since literal meanings may sometimes contribute to the construction of figurative interpretations, in the figurative-biasing context, the incompatible literal meaning may remain active even after the contextually appropriate figurative interpretation has been determined, as long as it is in some way supportive of the figurative interpretation. On the other hand, in the literally biasing context, where the contextually appropriate meaning is the literal one, the incompatible figurative meaning is usually irrelevant to the construction of the literal utterance meaning and hence becomes quickly suppressed.

Suppression of the contextually incompatible meaning postulated by Giora (2002) in the retention hypothesis is congruent with the suppression mechanism posed to play a crucial role in the understanding of figurative language by Gernsbacher and Robertson (1999), who define suppression as *a general cognitive mechanism, the purpose of which is to attenuate the interference caused by the activation of extraneous, unnecessary, or inappropriate information* (Gernsbacher and Robertson, 1999, p. 1619). Suppression has been experimentally demonstrated to attenuate inference during lexical access of ambiguous words, the processing of anaphoric and cataphoric reference, syntactic parsing, as well as in the understanding of metaphorical language (e.g., Gernsbacher and Robertson, 1999; Gernsbacher et al., 2001; Glucksberg et al., 2001).

Predictions of the retention hypothesis were tested by Giora and Fein (1999) in a word fragment completion test, which measured the amount of activation of literal and figurative meanings in literally and figuratively biasing contexts. Participants were first presented with short stories, ending with the target figurative sentence and then completed fragmented words which were related to either the literal or the figurative meaning of the target sentence. For each target sentence, two short texts were constructed, one biasing the literal meaning of the figurative target and the other biasing its metaphorical interpretation. For example, the literal biasing context for the target *Only now did they wake up* was a story about people partying and dancing all night, and some people calling on their friends the day after the party with the friends opening the door half asleep. In turn, the metaphorically biasing context for the same target sentence was a story about a bloody war going on in central Europe in which thousands of innocent lives had been lost before a decision was made to intervene and put an end to the massacres. In accordance with the authors’ predictions, familiar metaphorical expressions activated both their literal and figurative meanings in both types of context, with the figurative meaning retained to a significantly smaller extent in the literally than in the figuratively biasing context. According to Giora and Fein (1999), these results supported the view that in the literally biasing context with which it is incompatible, the non-literal meaning of a figurative expression gets suppressed very quickly, whereas in the figuratively biasing context the literal meaning of a figurative expression is retained because of its relevance for utterance processing. Overall, results reported in the study described here are compatible with the retention hypothesis, as both literal and figurative meanings were found active in dominant and balanced bilinguals, and literal meanings were retained even in contextually incompatible figurative-biased utterances.

What can existing models say about bilingual metaphoric processing? Although previous findings (e.g., Nelson, 1992; Vaid et al., 2015) have found evidence that bilinguals, like monolinguals, have direct access to the metaphor’s figurative interpretation, as hypothesized by Direct Access Models, the present results are more consistent with Giora’s (2003) GSH and Cieślicka’s (2006, 2015) Literal Salience Model. As revealed by the two-way interaction for the first fixation duration reading measure, language dominance moderates which meaning (literal vs. figurative) is more salient, and as argued by Cieślicka (2006, 2015), there is a propensity for the literal meaning of metaphoric expressions to be more readily accessible and more salient for bilingual speakers.

## Author Contributions

Both authors have made substantial, direct and intellectual contribution to the work, and approved it for publication.

## Conflict of Interest Statement

The authors declare that the research was conducted in the absence of any commercial or financial relationships that could be construed as a potential conflict of interest.
